# Phytochemical Analysis and *In Vitro* Antimicrobial and Free-Radical-Scavenging Activities of the Essential Oils from *Euryops arabicus* and *Laggera decurrens*

**DOI:** 10.3390/molecules16065149

**Published:** 2011-06-21

**Authors:** Ramzi A. Mothana, Mansour S. Alsaid, Nawal M. Al-Musayeib

**Affiliations:** 1 Department of Pharmacognosy, College of Pharmacy, King Saud University, P.O. BOX 2457, Riyadh 11451, Saudi Arabia; Email: msalsaid@ksu.edu.sa (M.S.A.); nalmusayeib@ksu.edu.sa (N.M.A.-M.); 2 Department of Pharmacognosy, Faculty of Pharmacy, Sana’a University, Sana’a, Yemen

**Keywords:** essential oils, *Euryops arabicus*, *Laggera decurrens*, antimicrobial, antioxidant, Yemen

## Abstract

The essential oils of the aerial part of two Asteraceae species, namely *Euryops arabicus* Steud. and *Laggera decurrens* (Vahl.) Hepper and Wood, were studied by GC and GC/MS. In parallel the antimicrobial and antioxidant activities were evaluated. The investigation led to the identification of 48 and 44 compounds for both plants, respectively. The essential oil of *E. arabicus* was rich in oxygenated sesquiterpenes (39.9%). The oil also contained a high content of sesquiterpene hydrocarbons (24.1%). Compounds such as caryophyllene oxide (8.6%), T-cadinol (7.0%), spathulenol (5.2%), (*E*)-β-caryophyllene (6.0%) and 2-*epi*-(*E*)-β-caryophyllene (6.0%) were the main constituents of the oil. Oxygenated monoterpenes also predominated in *L. decurrens* (46.3%). The thymoquinone-derivative, 3-methoxy-2-methyl-5-(1-methylethyl)-2,5-cyclohexadiene-1,4-dione (28.1%), thymol (5.7%) and eudesma-11-en-4a-ol (7.0%) were the most abundant constituents. Both essential oils showed antimicrobial activity with MIC-values between 0.13–5.25 mg/mL. Furthermore, only the essential oil of *L*. *decurrens* exhibited a strong antioxidant activity (91%) at 500 µg/mL.

## 1. Introduction

Aromatic plants are frequently used in traditional medicine and essential oils and volatile constituents extracted from them are widely used as antioxidants and antimicrobial agents as well as for the prevention and treatment of different human diseases [1]. Currently, essential oils are attracting increasing interest in the scientific community and there is much research being performed on their pharmacological activities, particularly their antimicrobial and antioxidant properties, which are important for food preservation and the treatment of diseases of microbial and oxidative stress origin such as bacterial and viral infections, inflammations, cancers and cardiovascular diseases, including atherosclerosis and thrombosis.

The genera *Euryops* and *Laggera* belong to the family Asteraceae. The genus *Euryops* was revised by Nordenstam, who recognized 97 species [2,3,4], whereas the genus *Laggera* is a small genus of about 20 species, mainly found in tropical Africa and Southeast Asia [5].

The genus *Euryops* is represented by only one species growing in Yemen, which is *Euryops arabicus* Steud [6,7]. The plant is a small erect undershrub of up to 1 m height. It is used in Yemen and Saudi Arabia to heal wounds [7]. The genus *Laggera* growing in Yemen constitutes three species including *Laggera decurrens* (Vahl.) Hepper and Wood, this is an erect, much-branched aromatic plant species of up to 1 m height [6]. The plant is employed as traditional herbal medicine in the treatment of rheumatism and wounds.

In the course of our chemical and biological investigations on natural essential oils of plants growing in Yemen and their possible antimicrobial and antioxidant activities, the aims of this study were: (1) to investigate the chemical composition of the essential oils from the two traditionally used Asteraceae species *Euryops arabicus* Steud. and *Laggera decurrens* (Vahl.) Hepper and Wood using GC and GC/MS; (2) to investigate *in vitro* the antimicrobial activity of the oils by the determination of MIC values using the broth micro-dilution assay; (3) to determine *in vitro* the antioxidant activity of the oils by measuring the scavenging activity of the DPPH radical.

## 2. Results and Discussion

Hydrodistillation of the aerial parts of *Euryops arabicus* and *Laggera decurrens* afforded yellow oils with a yield of 0.35 and 0.70% (w/w) on dry weight basis, respectively. The retention indices, percentage composition and identification methods are given in Table 1, where the identified components are listed in order of their elution on the CP-Sil 5 CB column. The GC-MS analysis led to the identification of 48 and 44 constituents representing 93.5% and 91.9% of the total oil of *E*. *arabicus* and *L. decurrens*, respectively. Reviewing the available current literature, nothing was found concerning the qualitative and quantitative analysis of the essential oil of *L*. *decurrens*. The existing knowledge about *E. arabicus* is very limited.

In the oil of *E*. *arabicus* oxygen-containing sesquiterpenes (39.9%) predominated over sesquiterpene hydrocarbons (24.1%). The main components were caryophyllene oxide (8.6%), T-cadinol (7.0%), spathulenol (5.2%), (*E*)-β-caryophyllene (6.0%) and 2-*epi*-(*E*)-β-caryophyllene (6.0%).

The oil of *L*. *decurrens* was also characterized by a high percentage of oxygenated monoterpenes (46.3%, Table 1). Among them, 3-methoxy-2-methyl-5-(1-methylethyl)-2,5-cyclohexadiene-1,4-dione (3-methoxythymoquinone) (28.1%) and thymol (5.7%) were the most abundant. Moreover, oxygenated sesquiterpenes accounted for 22.7% of the total oil, with eudesma-11-en-4a-ol (7.0%), T-cadinol (5.1%) and caryophyllene oxide (3.4%) as the main compounds.

**Table 1:** Chemical composition of the essential oils of *E. arabicus* (A) and *L. decurens* (B).

Number	Compounds	RI	% A	% B	Identification
1	Tricyclene	923	0.2	-	1,2
2	α-Pinene	932	1.0	-	1,2,3
3	β -Pinene	975	0.4	-	1,2,3
4	α-Terpinene	1011	0.8	-	1,2
5	Limonene	1023	0.3	-	1,2,3
6	γ-Terpinene	1050	0.4	-	1,2,3
7	Fenchone	1070	1.1	0.3	1,2,3
8	Linalool	1084	3.0	0.4	1,2,3
9	α-Fenchol	1101	2.9	0.7	1,2,3
10	Exo-Fenchol	1109	0.4	-	1,2
11	Camphor	1124	5.8	2.3	1,2,3
12	Borneol	1152	0.4	0.2	1,2,3
13	Terpinen-4-ol	1164	1.0	-	1,2,3
14	α-Terpineol	1175	1.1	0.2	1,2,3
15	Myrtenal	1181	0.8	-	1,2,3
16	Thymoquinone	1215	-	0.5	1,2
17	Geraniol	1235	-	0.6	1,2,3
18	Thymol	1272	-	5.7	1,2,3
19	Carvacrol	1282	-	2.7	1,2,3
20	Dihydroedulan II	1285	0.4	-	1,2
21	Thymolacetate	1329	-	3.9	1,2
22	3-Methoxy-2-methyl-5-(1-methylethyl)-2,5-cyclohexadiene-1,4-dione	1364	-	28.1	1,2
23	α-Copaene	1379	0.8	-	1,2
24	African-2-ene	1388	1.0	-	1,2
25	Modheph-2-ene	1392	-	2.0	1,2
26	β–Isocomene	1417	-	0.4	1,2
27	(*E*)-β-Caryophyllene	1425	6.0	0.7	1,2,3
28	Massoia lactone	1442	-	0.7	1,2
29	(*E*)-β-Farnesene	1445	1.5	-	1,2
30	α-Humulene	1457	0.8	0.4	1,2
31	β-Ionene	1464	0.3	0.2	1,2
32	2- *epi*-(*E*)-β-Caryophyllene	1470	6.0	-	1,2
33	γ-Muurolene	1476	-	0.2	1,2
34	Germacrene D	1481	1.1	-	1,2
35	Eremophilene	1487	1.7	-	1,2
36	β-Selenine	1490	-	2.3	1,2
37	γ-Patchoulene	1497	1.0	-	1,2
38	α-Selenine	1499	-	0.8	1,2
39	β-Curcumene	1503	-	0.4	1,2
40	γ-Cadinene	1512	-	0.3	1,2
41	δ-Cadinene	1518	2.7	-	1,2
42	7- *epi*-α-Selinene	1520	-	0.5	1,2
43	α-Calacorene	1534	1.2	-	1,2
44	(*E*)-Nerolidol	1549	1.9	0.6	1,2
45	*n*-Dodecanoic acid	1556	1.0	1.1	1,2
46	Spathulenol	1574	5.2	1.3	1,2
47	Caryophyllene oxide	1581	8.6	3.4	1,2,3
48	Humulene epoxide I	1592	1.0	0.6	1,2
49	β-Oplopenone	1598	3.9	-	1,2
50	Humulene epoxide II	1604	1.2	1.4	1,2
51	γ-Eudesmol	1618	1.6	1.7	1,2
52	T-Cadinol	1633	7.0	5.1	1,2
53	α-Cadinol	1647	4.1	-	1,2
54	Eudesm-11-en-4a-ol	1656	2.0	7.0	1,2
55	8α-Hydroxyeudesma-3,11-diene	1665	1.9	1.6	1,2
56	Amorpha-4,9-diene-2-ol	1676	1.5	-	1,2
57	*n*-Heptadecane	1697	0.8	0.8	1,2
58	*n*-Tetradecanoic acid	1744	1.0	1.5	1,2
59	6,10,14-Trimethy-lpentadecan-2-one	1827	1.6	2.2	1,2
60	Neophytadiene	1834	0.8	-	1,2
61	3,6,9-Nonadecatriene	1868	0.8	-	1,2
62	Hexadecanoic acid	1947	2.7	5.5	1,2
63	Cemberene A	1968	-	0.4	1,2
64	Manool	2060	-	0.9	1,2
65	Phytol	2100	0.8	0.1	1,2
66	Linoleic acid	2118	-	0.8	1,2
67	*n*-Pentacosane	2496	-	0.6	1,2
68	Heptacosane	2696	-	0.8	1.2
	Monoterpene hydrocarbons		2.9	0.0	
	Oxygenated monoterpenes		16.9	46.3	
	Sesquiterpene hydrocarbons		24.1	8.2	
	Oxygenated sesquiterpenes		39.9	22.7	
	Diterpene hydrocarbons		0.8	0.4	
	Oxygenated diterpenes		0.8	1.0	
	Aliphatic acids		5.2	8.9	
	Other compounds		2.9	4.4	
	Total		93.5	91.9	

RI, retention indices relative to C8-C30 n-alkanes on the CP-Sil 5 CB column, 1: retention index, 2: mass spectrum, 3: spiking with authentic compound.

To our knowledge this work represents the first GC-MS analysis of the essential oil of *L*. *decurrens*. A previous study on the alcoholic extract of *L*. *decurrens* grown in Africa led to the isolation of two phytotoxic compounds, namely 3-hydroxythymoquinone and 5-acetoxy-2-hydroxythymol [8].

In earlier studies [9,10,11,12,13,14,15,16,17,18,19], the chemical composition of the essential oils of different *Laggera* species e.g., *L. aurita*, *L. alata*, *L. gracilis*, *L. oloptera*, *L. pterodonta* and *L. tomentosa*, was investigated and a completely different chemical composition profile was reported. The previous studies on the genus *Laggera* revealed that two chemotaxonomic groups could be established [9]. The first one represents species, e.g., *L.* *pterodonta* [14], *L. pterodonta* [16], *L. gracilis* [13], *L. alata* [11] and *L. alata* var. *montana* [13] harvested from different African countries. The chemical composition of the oils showed the predominance of the phenolic ether 2,5-dimethoxy-*p*-cymene together with sesquiterpenes, e.g., β-caryophyllene, α-humulene and α-muurolene or oxygenated sesquitepenes, e.g., γ-eudesmol and α-eudesmol. The second chemotaxonomic group constitutes species that are completely devoid of the phenolic ether 2,5-dimethoxy-*p*-cymene, e.g., *L. tomentosa* from Ethiopia in which main volatile oil constituents are oxygenated monoterpenes e.g., chrysanthenone [18,19] and *L. oloptera* from Burkina-Faso which affords mainly monoterpene hydrocarbons, e.g., α-pinene [13].

On the basis of the obtained results, *Laggera decurrens* from Yemen may constitute a third chemotaxonomic group with a unique chemical content represented by 3-methoxythymoquinone as a predominant constituent.

The results of the antimicrobial activity are shown in Table 2. It was shown that the oils had varying degrees of growth inhibition against the bacterial strains (MIC-values: 0.13 and 5.25 mg/mL). The Gram-positive strains showed more susceptibility to the tested essential oils than the Gram-negative ones. On the other hand, no activity was registered against *Candida albicans*. The essential oil of *L. decurrens* demonstrated the greatest activity where the lowest MIC values (0.13 mg/mL) were obtained against *Staphylococcus aureus* (Table 2). Oxygenated monoterpenes such as thymol, carvacrol, thymoquinone, camphor, borneol, linalool and α-terpineol, were reported to be responsible for the antimicrobial activity of several essential oils [20,21,22,23,24,25]. Thus, the antimicrobial activity of the two investigated oils could be attributed to the high percentage of oxygenated monoterpenes such as 3-methoxythymoquinone, thymol, camphor, carvacrol, linalool and α-fenchol.

**Table 2:** Free radical scavenging activity and antimicrobial activity (MIC-values) of the investigated essential oils.

Plant species	Radical scavenging activity in %					MIC ^a^				
	10 (µg/mL)	50 (µg/mL)	100 (µg/mL)	500 (µg/mL)	1000 (µg/mL)	*S.* *aureus*	*B.* *subtilis*	*E. coli*	*P.* * aeuginosa*	*C.* *albicans*
*E. arabicus*	8.4	10.5	11.2	24.7	34.0	0.65	0.32	5.25	5.25	-
*L. decurens*	12.8	29.1	65.6	91.4	93.1	0.135	0.27	4.37	4.37	-
Amoxicillin						3.5	3.5	nt	nt	nt
Gentamicin						nt	nt	3.5	7.0	nt
Nystatin						nt	nt	nt	nt	3.5
Ascorbic acid	48.2	89.5	95.8	96.1	96.0					

^a^: minimum inhibitory concentration values are given as mg/mL for essential oils and μg/mL for standard antibiotics, nt: not tested.

Moreover, the high percentage of some oxygenated sesquiterpenes such as caryophyllene oxide, T-cadinol, α-cadinol and eudesm-11-en-4a-ol might contribute to the observed strong activity and a possible synergistic activity should also be taken in consideration.

The potential antioxidant activity of the oils was determined on the basis of scavenging activity of the stable free radical DPPH. Only the oil of *L. decurrens* was able to reduce DPPH and to show antioxidant activity (Table 2). The oil showed at 500 µg/mL a strong antioxidant activity (91%) comparable with that of the ascorbic acid (96%). This observed effect is certainly associated with high content of phenolic components such as 3-methoxythymoquinone, thymol and carvacrol in the essential oil of *L. decurrens* [26,27,28,29].

## 3. Experimental

### 3.1. Plant Material

*Euryops arabicus* was collected from Soqotra Island in January 2007 while *Laggera decurrens* was collected from the Al-Mahwit district in May 2007. Both plants were identified at the Pharmacognosy Department, Faculty of Pharmacy, Sana'a University. Voucher specimens were deposited at the Pharmacognosy Department, Faculty of Pharmacy, Sana'a University.

### 3.2. Extraction of the Essential Oil

The collected, air-dried and ground aerial parts of both plants were submitted to hydrodistillation for 3 h using a Clevenger-type apparatus according to the European Pharmacopoeia. The obtained oils were dried over anhydrous sodium sulfate and after filtration, stored at +4 °C until tested and analyzed.

### 3.3. Gas Chromatography Analysis

The essential oils were analyzed on a Hewlett Packard GC (5890 Series II) equipped with a Flame Ionization Detector (FID). The analysis was carried out on a fused silica capillary CP-Sil 5 CB column (Varian, 30 m × 0.25 mm i.d., film thickness 0.26 μm). Nitrogen was used as a carrier gas at a flow rate of 0.7 mL/min. Injector and detector temperature were set at 250 °C and 280 °C, respectively. Oven temperature was kept at 45 °C then gradually raised to 280 °C at 3 °C/min and finally held isothermally for 22 min. Diluted samples (1/100 in pentane, v/v) of 1.0 μL were injected manually (split mode, split ratio 1:16). Calculation of peak area percentage was performed on basis of the FID signal using the GC HP-Chemstation software (Agilent Technologies).

### 3.4. Gas Chromatography-Mass Spectrometry

The GC-MS analyses were conducted on a Hewlett-Packard 5890 series II gas chromatograph coupled to a VG Analytical 70-250S mass spectrometer. The GC was equipped with a fused silica capillary CP-Sil 5 CB column (25 m × 0.25 mm i.d., film thickness 0.4 μm, from Chrompack, Varian). Helium was used as carrier gas at a flow rate of 1 ml/min. Injector temperature was 200 °C. Oven temperature was programmed from 80 °C (2 min hold) at 10 °C/min to 270 °C and finally held isothermally for 20 min. For GC-MS detection, an election impact ionization system, with ionization energy of 70 eV was used. A scan rate of 0.6 sec (cycle time: 0.2 second) was applied, covering a mass range from 35 to 600 *m/z*.

### 3.5. Identification of Components

The identification of the constituents was achieved by the comparison of their retention indices and mass spectra with data generated under identical experimental conditions. Therefore, special software was applied with a library integrated which used a two-dimensional search algorithm considering the retention index as well as mass spectral similarity [30]. In addition, MassLib (V9.3-106) for processing and interpretation of mass spectra (MassLib, 1996–2008) was used with several commercially available libraries included Wiley Registry of Mass Spectral Data (4th edition), NIST/EPA/NIH Mass spectral Library (2005), Library MPI Mühlheim (2006), Geochemicals (1st edition), MRC collection (1st edition), and CC (4th edition)—All from Chemical Concepts (Wiley). As an additional library the electronic MS data base of Adams was used [31]. Moreover, the comparison was achieved with authentic reference compounds available in our laboratories. The retention indices were determined in relation to a homologous *n*-alkanes series (C_8_-C_30_) under the same operating conditions. Components relative concentrations were obtained by peak area normalization. No response factors were calculated.

### 3.6. Determination of Antimicrobial Activity

#### 3.6.1. Test organisms

The following microorganisms were used as test organisms in the screening: *Staphylococcus aureus* (BNI 18), *Bacillus subtilis* (BNI 28), *Escherichia coli* (BNI 2), *Pseudomonas aeruginosa* (BNI 20) and *Candida albicans* (BNI 33). The microbial strains were obtained from the Bernhard- Nocht Institute (BNI) for Tropical Medicine, Hamburg, Germany.

### 3.6.2. Broth micro-dilution assay for minimum inhibitory concentrations (MIC)

The broth micro-dilution method described by Mann and Markham [32] with modifications was used to determine the MIC of the investigated essential oils against the above mentioned microbial strains. With sterile round-bottom 96-well plates, duplicate two-fold serial dilutions of extract (100 μL/well) were prepared in the appropriate broth containing 5% (v/v) DMSO. Two-fold dilutions of amoxicillin, gentamicin or nystatin were used as a positive control. A bacterial cell suspension (prepared in the appropriate broth) of 100 μL, corresponding to 1 × 10^6^ CFU/mL, was added in all wells except those in columns 10, 11 and 12, which served as saline, essential oil and media sterility controls, respectively. Controls for bacterial growth without essential oil were also included on each plate. The final concentration of bacteria in the assay was 5 × 10^5^ CFU/mL. Plates were then incubated at 37 °C for 18 h overnight. After incubation, the MIC of each essential oil was determined as the lowest concentration at which no growth was observed in the duplicate wells. Twenty microliters of a *p*-iodonitrotetrazolium violet solution (0.04%, w/v) (Sigma, USA) was then added to each well. The plates were incubated for a further 30 min, and estimated visually for any change in color from yellow to pink indicating reduction of the dye due to bacterial growth. The highest dilution (lowest concentration) that remained yellow corresponded to the MIC. Experiments were performed in duplicate.

### 3.7. Determination of Antioxidant Activity (Scavenging Activity of DPPH Radical)

The DPPH free radical scavenging assay was carried out for the evaluation of the antioxidant activity. This assay measures the free radical scavenging capacity of the investigated extracts. DPPH (2,2-diphenyl-1-picrylhydrazyl) is a molecule containing a stable free radical. In the presence of an antioxidant which can donate an electron to DPPH, the purple color, typical for free DPPH radical decays, and the change in absorbency at λ = 517 nm is followed specrophotometrically. This test provides information on the ability of a compound to donate a hydrogen atom, on the number of electrons a given molecule can donate, and on the mechanism of antioxidant action. The method was carried out as described previously [33]. The essential oils were dissolved in methanol, respectively, and various concentrations (10, 50, 100, 500 and 1000 μg/mL) of each oil were used. The assay mixture contained in a total volume of 1 mL, 500 μL of the oil, 125 μL prepared DPPH (1 mM in methanol) and 375 μL solvent (methanol). After 30 min incubation at 25 °C, the decrease in absorbance was measured at λ = 517 nm. The radical scavenging activity was calculated from the equation:

% of radical scavenging activity = Abs_control_ − Abs_sample_ / Abs_control_ × 100

## 4. Conclusions

In conclusion, the obtained data indicate that the essential oils of *E*. *arabicus* and *L*. *decurrens* exhibit potent antibacterial activity, which support their use in traditional medicine in the treatment of wounds. The results clearly show that the oil of *L*. *decurrens* presents antioxidant activity and might be useful for therapeutic purposes to prevent ROS disorders. The biological activities could be partly explained by the presence of oxygenated monoterpenes, such as 3-methoxythymoquinone, thymol, camphor, carvacrol and linalool. Moreover, based on the high percentage of 3-methoxythymoquinone characterized in the oil of *L*. *decurrens*, a third chemotaxonomic group of the *Laggera* species could be established.
